# Prevalence, Distribution, and Risk Factors of Mycobacterium Other Than Tuberculosis Among Tuberculosis Presumptive Patients in Karonga District in Malawi

**DOI:** 10.1111/crj.70126

**Published:** 2025-09-18

**Authors:** S. Chitsulo, L. Gogoda, H. Nyirenda, S. Chirwa, T. Mwenyenkulu, H. Kanyerere, J. Mpunga, K. Mbendera, B. Mbakaya, S. Mwale, B. Nyambalo, F. Sinyiza, M. Chisale

**Affiliations:** ^1^ Malawi Ministry of Health, National TB and Leprosy Elimination Program Lilongwe Central Region Malawi; ^2^ Faculty of Science Technology and Innovation Mzuzu University Mzuzu Northen Region Malawi; ^3^ Mzuzu Central Hospital Mzuzu Northern Region Malawi

**Keywords:** MOTT, mycobacterium, presence, presumptive

## Abstract

**Introduction:**

Besides tuberculosis (TB), there are also other nontuberculous mycobacteria (NTM) that present with similar clinical signs and symptoms as TB. If not promptly found and treated, these organisms may affect the programming of the TB control and elimination campaign. The study sought to establish the prevalence, distribution, and factors contributing to MOTT infections among presumptive TB patients in the Karonga district.

**Methods:**

A descriptive cross‐sectional study research design was employed. A total of 196 participants were included in the study using a census approach. Data were collected by administering a questionnaire to the health care worker, and a sputum specimen was collected from the participants; this specimen was used to examine the presence of mycobacterium using the microscope. Regardless of the results at the district‐level laboratory, all the specimens were then sent to the Mzuzu region TB reference laboratory to isolate *Mycobacterium tuberculosis* and Mycobacterium Other Than Tuberculosis.

**Results:**

Of the 196 samples collected, 14 (7.1%) were positive at the district level. When sent for culture, 195 (99.5%) had culture results, and 23 (12%) had growth in culture. Out of the 23 (100%) culture‐positive results, 12 (52%) were MOTT‐positive, while 11 (48%) were MTB complex. There were more men, seven (58%) with MOTT‐positive than women, five (42%), and more in the age group of 15–39 years old, with six (50%) and less in more than 60 years old two (16.7%).

**Conclusion:**

The results show the presence of MOTT infections among presumptive TB patients who submitted samples to the study. The distribution by sex shows that more men had MOTT infections than women. However, all the risk factors listed for the study were not significant for MOTT infections. The recommendation is to improve the testing techniques to identify these microorganisms, which are neglected but very difficult to assess, especially when no clear population is at risk of getting these infections compared with TB.

## Graphical abstract

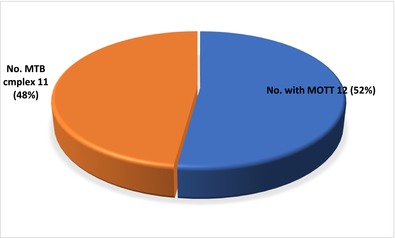

## 1 Introduction

Mycobacterium other than tuberculosis (MOTT) is one of the public health problems affecting the lungs, and these infections are rapidly increasing worldwide [1, 2]. These infections are not in the notifiable disease category in most countries; it is, therefore, difficult to notice their availability compared with others, such as TB, though they present the same signs and symptoms of TB; hence, it is difficult to differentiate them clinically [3, 4]. This is equally the same in diagnosing MOTT because of the high burden of TB. Every positive smear microscopy indicates MTB, and the patient is started on TB treatment. The molecular mycobacterium assay definition that Malawi is currently using does not detect mycobacterium outside the MTB complex. Hence, it is likely to be reported as negative when the client is truly positive for MOTT if the facilities use these molecular test methods as the first line in testing presumptive clients. However, another study on the presence of NTM in the northern part of Malawi among MDR‐TB suspected patients found that 18% of presumptive MDR TB patients had MOTT [5, 6, 7]. This percentage is high compared with other findings from different prevalence studies in countries such as Germany, India, Nigeria, and Zambia, where the prevalence was between 6% and 15%. It is essential to note that Karonga District is in the northern part of Malawi; therefore, it is more likely that the 18% found includes patients from Karonga District.

More information needs to be provided in Malawi about the incidence and prevalence of MOTT, which further challenges the knowledge of distribution and risk factors that cause MOTT in the population. Therefore, conducting this study to establish the presence, distribution, and factors contributing to MOTT in Karonga was of great importance.

## 2 Methods

Study Settings: The study was conducted in urban and rural residences in the Karonga district.

Study Design: A cross‐sectional study using a census approach was used to select all pulmonary presumptive TB patients for 2 months.

Sample Size and Sampling Technique: Since it was a census approach, all presumptive TB patients were recruited within the data collection period.

Ethical Approval: The National Health Sciences Research Committee granted ethical approval to ensure the safety and protection of the participants: ethical approval number 23/01/3149. Before commencing data collection, participants were provided with study information, and consent was obtained from all clients involved.

Recruitment Methods: All TB presumptive clients coming for TB diagnosis were taken as participants after consented to participate in the study. For each participant, we collected demographic, social, and clinical history by reviewing the participants' health passport book, especially on their HIV status and whether the participants had some Communicable or Non‐Communicable Diseases (NCD). Social data were collected by asking whether the participant had a history of taking alcohol, smoking cigarettes, and traveling outside the country. This information was collected using a questionnaire, and all participants submitted sputum samples for examination using microscopy at the district hospital laboratory. The sputum samples collected were checked to meet the quality sample collection, handling, and storage criteria, which included the volume of 3–5 mL, a well‐completed laboratory request form that matches the information on the sample collection container, well‐packed using triple packaging standards and transferred within 4 days of collection to the Mzuzu region TB reference laboratory.

Data Collection Procedures: This study was specifically looking for the prevalence, distribution, and factors contributing to MOTT among presumptive TB clients. Therefore, social demographic data and clinical history were collected using a questionnaire administered by a trained healthcare worker (research assistant), including the participant's clinical history from the health passport books and treatment cards. Samples of these participants were collected from all the health facilities within the Karonga district. These samples were transferred from the health facility to the district laboratory by riders for health (R4H) courier services, where testing was done using microscopy. Microscopy is one method used for the diagnosis of mycobacterium species, including TB, in poor countries because it is easy and convenient, though proven to have a low sensitivity of 20% to 70% with a specificity of 95%–98% depending on how competent the Microscopist is [8]. Microscopy was used to detect all mycobacterium species holding the first stain after exposure to the decoloring solution acid‐alcohol because they have acid‐fast characteristics and give a contrasting background after the counter‐stain [9]. The same samples were sent to the Mzuzu TB reference laboratory for culture using the Mycobacteria Growth Indicator Tube (MGIT) and line probe assay (LPA) [10].

Statistical Analysis: Descriptive data analysis was utilized to analyze quantitative data generated from the study subject through interviews and laboratory results. A computer software package, Statistical Package for Social Sciences (SPSS), was utilized to compute the frequencies and percentages for descriptive analysis.

## 3 Results

### 3.1 Social and Demographic Characteristics of Clients

Among the 196 (100%) participants that were registered in the study, 108 (55.1%) were males with the highest age range of 40–59 years, 71 (36.2%), and the most minor, six (3.1%) were between 0 and 4 years. Most participants, 139 (70.9%), resided in rural areas, while 57 (29.1%) resided in urban areas. On education levels, the majority, 132 (67.3%) of the participants, had attended primary school education, while four (2.0%) had no education. About marital status, 146 (74.5%) clients were married. A good proportion of clients, 96 (49%) registered, mentioned farming as their primary source of income, while one (0.5%) was a miner. Most (85.7%) of the participants had not stayed outside Malawi, whereas 28 (4.3%) had stayed outside the country, most of which 12 (6.1%) mentioned Tanzania as a country of residence. (Table 1).

**TABLE 1:** Demographic characteristics of clients.

Characteristics		Frequency: *n* (%), *N* = 196
Age group	0–14 15–39 40–59 60 above	6 (3.1) 70 (35.7) 71 (36.2) 49 (25.0)
Sex	Male	108 (55.1)
	Females	88 (44.9)
Residence	Urban	57 (29.1)
	Rural	139 (70.9)
Education background	No Education	4 (2.0)
	Primary	132 (67.3)
	Secondary	55 (28.1)
	Tertiary	5 (2.6)
Marital status	Married	146 (74.5)
	Not married	50 (25.5)
Occupations	Architecture	15 (7.7)
	Administrative	6 (3.1)
	Health worker	3 (1.5)
	Miner	1 (0.5)
	Farmer	96 (49.0)
	Business	33 (16.8)
	Housewife/husband	16 (8.2)
	Others	26 (13.3)
Ever stayed outside Malawi?	Yes	28 (14.3)
	No	168 (85.7)
Which country	South Africa	4 (2)
	Tanzania	12 (6.1)
	Zambia	10 (5.1)
	DRC	2 (1.0)
	Not traveled	168 (85.7)

### 3.2 Clinical Characteristics of Clients

Out of the total, 112 (57.15) of the participants had no HIV, while 77 (39%) of the clients registered in the study were HIV positive, and seven (3.6%) had an unknown status. Regarding the history of having taken TB medication before, 176 of 196 (89.8%) had never taken TB medication before, and 20 (10.2%) had taken TB medication before. A majority of the participants, 125 (63.8%), were not on any drug during the study, while 71 (36.2%) were on other medications, most of which 63 (32%) were on Anti‐Retroviral Therapy (ART). One hundred sixty‐five participants (84.2%) had never stayed with a TB patient, while only 27 (13.8%) had. On alcohol intake, 113 (57.7%) of the clients had no history of alcohol consumption. With regard to smoking, 154 (78.6%) mentioned having never smoked, and a high number of participants, 180(91.8%), had no other health conditions, 10 (5.1%) had other health conditions, four (2.0%) had Chronic Obstructive Pulmonary Disease (COPD)/Asthma, and two (1.0%) had hypertension (Table 2).

**TABLE 2:** Clinical characteristics of the clients.

Health characteristics		*n* (%), *N* = 196
HIV status	Positive	77 (39.3)
	Negative	112 (57.1)
	Not known	7 (3.6)
History of TB drugs	Yes	20 (10.2)
	No	176 (89.8)
Taking any other drugs?	Yes	71 (36.2)
	No	125 (63.8)
Name of drug	ART	63 (32.1)
	Other	10 (5.1)
	Not on any drug	123 (62.8)
Ever stayed with a TB patient	Yes	27 (13.8)
	No	165 (84.2)
	Do not know	4 (2.0)
Ever taken alcohol?	Yes	83 (42.3)
	No	113 (57.7)
Ever smoked cigarette	Yes	42 (21.4)
	No	154 (78.6)
Any health conditions	No	180 (91.8)
	COPD/asthma	4 (2.0)
	Hypertension	2 (1.0)
	Others	10 (5.1)

### 3.3 Laboratory Findings

**FIGURE 1:**
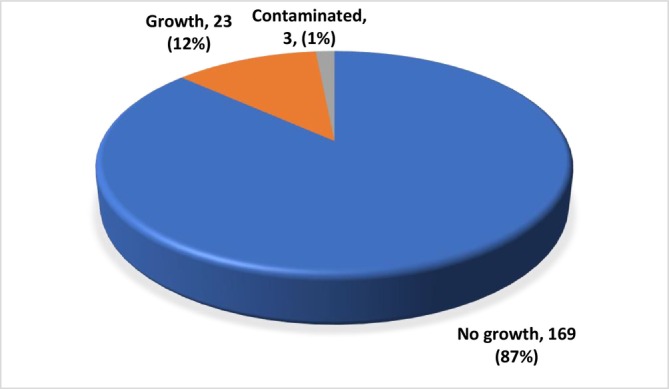
MIGT laboratory results.

Of 196 samples collected, 14 (7.1%) were positive at the district level, and of the 195 samples tested at the culture laboratory, 169 (87%) had no growth, and 23 (12%) had mycobacterium growth. Among the results, three (1%) were contaminated cultures, as shown in Figure 1.

**FIGURE 2:**
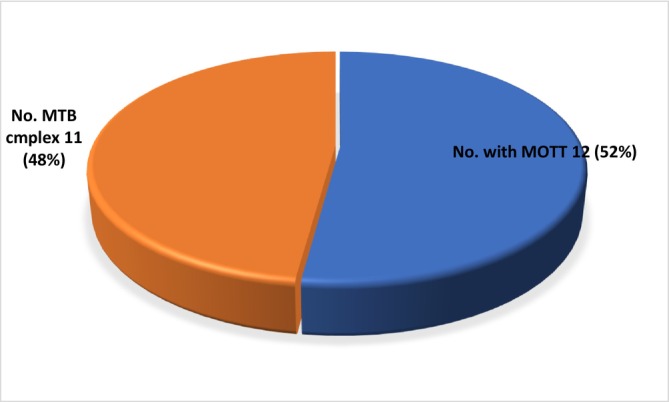
Differentiation of mycobacterium.

Among the 23 samples that had growth, 11 (48%) were isolated to have MTB complex, while 12 (52%) MOTT were isolated (Figure 2).

### 3.4 Risk Factors Contributing to MOTT Among Presumptive TB Clients

Statistical tests on risk factors contributing to MOTT did not identify any attribute that is significantly associated with MOTT (Table 3).

**TABLE 3:** Risk factors contributing to MOTT among presumptive TB clients.

Risk factor	*N* = 196 (%)		*p*
Smoking	MOTT	No MOTT	
Smoked	4 (33.3)	38 (20.7)	0.300
Never smoked	8 (66.7)	146 (79.3)	
Alcohol intake			
Ever taken alcohol	5 (41.7)	78 (42.4)	0.961
Never taken alcohol	7 (58.3)	106 (57.6)	
HIV status			
Positive	5 (41.7)	72 (39.1)	0.624
Negative	6 (50.0)	106 (57.6)	
Unknown	1 (8.3)	6 (3.3)	
Resided outside Malawi			
Yes	1 (8.3)	27 (14.7)	0.453
No	11 (91.7)	157 (84.3)	
Residence in specific countries			0.878
South Africa	0 (0)	4 (2.2)	
Tanzania	1 (8.3)	11 (6.0)	
Zambia	0 (0)	10 (5.4)	
DRC	0 (0)	2 (1.1)	
Not travelled	11 (91.7)	157 (85.3)	
Other health conditions			0.353
No condition	11 (97.7)	169 (91.8)	
COPD/asthma	1 (8.3)	3 (1.6)	
Hypertension	0 (0)	2 (1.1)	
Other	0 (0)	10 (5.4)	
Sex of client			0.816
Male	7 (58.3)	101 (54.9)	
Female	5 (41.7)	83 (45.1)	
Age of client			0.681
0–14 years	0 (0)	6 (3.3)	
15–39 years	6 (50)	64 (34.8)	
40–59 years	4 (33.3)	67 (36.4)	
60 years and above	2 (16.7)	47 (25.5)	
Area of residence			0.322
Urban	5 (41.7)	52 (28.3)	
Rural	7 (58.3)	132 (71.7)	
Education background			0.864
No education	0 (0)	4 (2.2)	
Primary	9 (75.0)	123 (66.8)	
Secondary	3 (25.0)	52 (28.3)	
Tertiary	0 (0)	5 (2.6)	
Marital status			0.521
Married	8 (66.7)	138 (75.0)	
Not married	4 (33.3)	46 (25.0)	
Source of income			0.753
Construction	0 (0)	15 (8.2)	
Administrative	0 (0)	6 (3.3)	
Health worker	0 (0)	3 (1.6)	
Miner	0 (0)	1 (0.5)	
Farmer	8 (66.7)	88 (47.8)	
Business	3 (25.0)	30 (16.3)	
House wife/husband	0 (0)	16 (8.7)	
Other	1 (8.3)	25 (13.6)	
History of TB drugs			0.825
Yes	1 (8.3)	19 (10.3)	
No	11 (91.7)	165 (89.7)	
Taking other drugs			0.404
Yes	3 (25.0)	68 (37.0)	
No	9 (75.0)	116 (63.0)	
Use of specific drugs			
ART	2 (16.7)	61 (33.2)	0.468
Other	1 (8.3)	9 (4.9)	
Not on any drug	9 (75.0)	114 (62.0)	
Staying with a TB patient			0.404
Yes	3 (25.0)	68 (37.0)	
No	9 (75.0)	116 (63.0)	

## 4 Discussion

This study discovered the 6.2% prevalence of MOTT infections among presumptive TB participants from the Karonga district. Since MOTT infections are among the neglected diseases, it is difficult to measure whether their prevalence is high or low since we have no national target percentage. However, several studies in various countries have indicated an increase in these infections from 2003 to 2016 [10]. Of the 195 (100%) samples analyzed, 12 (6.2%) were MOTT positive. These findings concur with the conclusions of the Nationwide Drug Resistance Tuberculosis Prevalence Survey of 2011, which found that among 768 previously treated TB cases, 19 (2.5%) were MOTT, while for 1347 new TB cases, six (0.4%) were MOTT. The study also revealed that of the six new MOTT patients, two were from Karonga district, providing further evidence that Karonga District has MOTT infections detected among the TB patients. These findings also agree with those of a study on the prevalence and distribution of NTM among MDR‐TB suspected patients in Northern Malawi, which discovered that out of 170 culture‐positive reviews, 30 (18%) were positive for MOTT of presumptive MDR TB patients. The findings of this study are, however, higher than those of the national prevalence study and less than the prevalence and distribution of NTM among MDR‐TB suspected patients in Northern Malawi. This could be because these two studies had a larger sample size with study subjects as MDR TB patients, which is different from this, which only included the presumptive TB participants from Karonga District.

Different studies have attributed the distribution of MOTT to various aspects, most of which pointed to the age and geographic diversity of the patient as being in play. Other studies discovered that people more than 35 and 39 years of age stood a high risk of developing NTM, especially in dusty seasons [11, 12]. Although the ages are different in these two studies, the idea that increasing age increases the risk of MOTT infections is the same. The study found that 3/100 000 had MOTT in the < 39‐year‐old population and 64/100 000 in the > 80‐year‐old population [13]. Their study indicated that the more years, the higher the risk of getting MOTT infections. This study, however, did not find any significant relationship between the age of the client and the manifestation of MOTT. The study found that 6/12 (50%) of MOTT cases were aged between 15 and 39 years, 4/12 (33%) between 40 and 59 years, and 2/12 (17%) above 60 years. This distribution was, however, not significant at a *p*‐value of 0.681. This difference could be attributed to differences in predisposing factors and differences in MOTT species, some of which affect individuals of all ages, for exmaple, *M. abscessus* in contrast, others are age‐specific, e.g., *M. gordonae* [14, 15].

Other studies also attribute MOTT infection distribution to the geographic location of an individual. A study on the global distribution of MOTT worldwide revealed that NTM prevalence and species vary across different countries and regions [16]. The study was done on all six continents but indicated that Africa (represented by South Africa) had a high prevalence of respiratory non‐tuberculous infections compared with other continents worldwide. This means that even within individual countries, regions, and districts, the distribution could differ due to different factors. A study conducted in South Korea showed that the distribution of MOTT differed within regions and districts of the same country. It also showed variations in infection rates within the same district, for example, between urban and rural [17]. This study did not, however, find a significant difference between urban and rural dwellers. The study revealed that of the 12 MOTT‐positive participants, seven (58.3%) were from rural areas, whereas five (41.7%) were from urban regions in Karonga. The difference was, however, not significant at a *p*‐value of 0.322. This sharply contrasts with the findings of a study conducted in Zambia, which found a higher prevalence of MOTT among rural dwellers than urban dwellers [12]. This difference could be due to differences in social patterns between rural and urban dwellers in Karonga and Zambia.

The study also assessed the relationship between MOTT infections and other factors that could put clients at risk, such as smoking, alcohol intake, HIV status, history of residence outside Malawi, other underlying health conditions, history of anti‐TB drugs, use of different medications, and history of contact with a TB patient. This study has revealed no significant differences between MOTT infections and these factors at *p*‐values of 0.3, 0.961, 0.624, 0.453, 0.353, 0.825, 0.404, and 0.404, respectively. The findings of this study are contrary to those of other studies conducted in both Malawi and outside. Some findings reported that people with lung damage previously treated pulmonary tuberculosis and emphysema, and children stand a higher risk of having MOTT infections [18]. This is also in agreement with another study, which stated that immunosuppression, such as in people living with HIV/AIDS, is at a higher risk of developing MOTT. The difference could be due to differences in the study setup and sample size.

## 5 Conclusion

The study has shown that the prevalence of MOTT is higher than the prevalence of tuberculosis among presumptive TB patients in Karonga. The study also found men to have these infections more than women, though the difference was insignificant. The findings of this study did not find any correlation between MOTT infections and any of the following risk factors: smoking, alcohol intake, HIV status, history of residence outside Malawi, other underlying health conditions, history of anti‐TB drugs, use of different medications, and history of contact with a TB patient. Therefore, these challenges are understanding and managing infections as you do not give the risk category to target. This brings an important area to monitor even in other non‐suggestive predisposing infections and factors to establish the ideal diagnosis for better clinical management to avoid worsening health conditions due to MOTT infection complications.

## Limitation of the Study

From the findings of this study, it is imperative to note that this review has some limitations; we did not describe much regarding the global epidemiology of Mycobacterium Other Than Tuberculosis, and this article may not be of particular interest to researchers outside Malawi. This study was intended to serve as a local epidemiologic reference for physicians and scientists in Malawi. Lastly, the study was only intended to ascertain the presence of MOTT among presumptive TB patients. It did not go further to differentiate the species of MOTT identified.

## Author Contributions

S.C. conceived the idea and participated in the design, data collection, data analysis, and interpretation of the data and developed and reviewed the manuscript. L.G. conceived the idea and participated in the design, data collection, data analysis, and interpretation of the data and developed and reviewed the manuscript. H.N. participated in data analysis and interpretation of the data. T.M. participated in the interpretation of the data and reviewed the manuscript. H.K. participated in the design and reviewed the manuscript. J.M. participated in data analysis and reviewed the manuscript. K.M. reviewed the manuscript. S.C. participated in data analysis and interpretation. M.C. participated in the design and data collection, conducted laboratory procedures, and reviewed the manuscript. B.M. participated in the interpretation of the data and reviewed the manuscript. S.M. participated in data analysis and interpretation of the data. F.S. participated in data collection, data analysis, and data interpretation and reviewed the manuscript. B.N. participated in the manuscript review.

## Ethics Statement

To achieve this, ethical approval was sought from the National Health Sciences Research Committee to ensure that the safety and protection of the participants are guaranteed.

The study observed the following ethical considerations:

Voluntary participation: The respondents' permission was sought before the researcher administered the questionnaire.

Informed consent: The respondents were informed of the study's objectives so that they could decide whether to participate in the research.

Anonymity: All information that was collected was kept confidential. The respondents were assigned aliases or codes instead of their names. All the analyzed information was generalized and not attached to any particular respondent.

The information obtained from these participants was kept confidential by the researcher until the final dissertation was published, and it was only accessible to those who would use it for the study.

## Conflicts of Interest

The authors declare no conflicts of interest.

## Data Availability

The data supporting this study's findings are available on request from the corresponding author.
